# Digested Human Colostrum Reduces Interleukin-8 Production in Induced Human Intestinal Epithelial Cells

**DOI:** 10.3390/nu14142787

**Published:** 2022-07-06

**Authors:** Yang Lyu, Yimin Chen

**Affiliations:** Margaret Ritchie School of Family and Consumer Sciences, University of Idaho, Moscow, ID 83844, USA; yanglv@umass.edu

**Keywords:** human milk, infant, intestine, inflammation

## Abstract

Little is known about the impact of human colostrum on infant intestinal health following digestion. The aim of this study was to compare the effect of digested versus undigested human colostrum on inflammation and cytotoxicity in human intestinal epithelial cells (Caco2BBe) stimulated with lipopolysaccharides (LPS) or tumor necrosis factor (TNF). Colostrum samples (days 2–8 postpartum) from ten mothers of preterm infant were applied. Caco2BBe cells were pretreated by digested or undigested colostrum before stimulation with LPS or TNF. The inflammatory response was determined by measuring the production of interleukin-8 (IL-8) from cells using enzyme linked immunosorbent assay (ELISA). Cytotoxicity was examined by measuring the release of lactate dehydrogenase (LDH) from the cells. Digested colostrum significantly reduced IL-8 production under LPS and TNF stimulation compared with undigested colostrum. Individual colostrum samples exhibited wide variance in the ability to suppress IL-8 production and cytotoxicity in Caco2BBe cells. In vitro-digested human colostrum suppressed an inflammatory response more than undigested human colostrum in an induced intestinal cell culture model.

## 1. Introduction

Necrotizing enterocolitis (NEC) is a devastating gastrointestinal (GI) disease that affects 5% to 10% premature infants, resulting in a mortality of 28% to 35% [1,2]. Infants with NEC suffer from severe inflammatory responses that can lead to an intestinal barrier breakdown, impaired mucosa, bacterial translocation, and systemic inflammatory response [3]. The administration of human milk in preterm infants has been shown to reduce the risk of NEC [4,5]. However, the bioactive functions of human milk in the context of NEC mechanisms are not fully understood. Colostrum is the preferred first human milk for feeding infants due to its high concentrations of bioactive components [6]. Since NEC occurs at the distal small intestine where full digestion has occurred, it is imperative to determine how digested colostrum impacts the small intestine for better translation to the infant physiologic environment. Currently, most studies still use undigested human milk or colostrum to identify their bioactive effects. The method of in vitro digestion provides a powerful way to mimic the infant digestion process in vivo and enables the release of many bioactive molecules, as well as circumventing ethical issues in research with infants [7,8]. Current literatures have majorly investigated the bioactive functions of individual human milk factors [9,10,11]. To our knowledge, there is scarce evidence on the bioactive potency of human colostrum from digestion. The aim of this study is to compare the effect of digested human colostrum versus their undigested counterparts on inflammation and cytotoxicity using the human intestinal epithelial cells.

## 2. Materials and Methods

### 2.1. In Vitro Digestion of Colostrum Samples

Ten colostrum samples (days 2–8 postpartum) from different mothers of preterm infants in a previous study were used for this work [12]. Human colostrum samples were collected under an Institutional Review Board (IRB)-approved human milk repository and immediately deidentified. Human colostrum samples were snap frozen in liquid nitrogen once collected and stored at −80 °C before the experiments. Aliquots of each colostrum sample were either subjected to an in vitro digestion that simulates the preterm infant gastrointestinal environment or remained undigested. The digestion process has been described in the previous study [12]. In brief, colostrum samples were diluted to a concentration of 8.25% (*v*/*v*) before digestion with pepsin (≥250 units mg^−1^) with hydrochloric acid (pH 4.0) at 37 °C for 30 min with agitation. The samples were kept under 37 °C for another 30 min with sodium bicarbonate (pH 6.0). Then samples were digested with pancreatin and bile salts (6.7 mM pancreatin, 29.4 mM bile salt, 0.01 M sodium bicarbonate, pH 7.0) at 37 °C for 2 h. A final heat deactivation at 90 °C for 15 min was applied. The final concentration of human colostrum was 6.36% (*v*/*v*).

### 2.2. Cell Culture

Human intestinal epithelial (Caco2BBe) cells (CRL-2102) were obtained from the American Type Culture Collection (ATCC; Manassas, VA, USA) and cultured at 37 °C in an atmosphere of 5% carbon dioxide (CO_2_) in Dulbecco’s Modified Eagle Medium (DMEM; Gibco, Waltham, MA, USA) supplemented with 10% (*v*/*v*) fetal bovine serum (Gibco, Waltham, MA, USA) and 1% (*v*/*v*) non-essential amino acids (Gibco, Waltham, MA, USA) in T-25 flasks (Nunc, Waltham, MA, USA). The cell culture medium was changed every 48 h. Cells were passaged every 6 days with 0.25% trypsin-ethylenediaminetetraacetic acid (EDTA) (Gibco, Waltham, MA, USA). For all experiments, cells were seeded with the density of 4 × 10^4^ cells cm^−2^, 500 µL per well on 48-well plates (Corning, New York, NY, USA) coated with type I collagen (Millipore, Burlington, MA, USA). The plate culture medium contained an additional 1% (*v*/*v*) penicillin-streptomycin (Gibco, Waltham, MA, USA) to prevent contamination. After seven days, when 100% confluency was reached, cells were cultured for another 21 days to reach full differentiation as to mimic properties of fetal epithelial cells [13].

### 2.3. Colostrum Treatment and Stimulation

Cells from passage 7–9 were used for this experiment. On Day 21, the maintenance cell culture medium was removed from the 48-well plates. Cells were pre-incubated with 2% (*v*/*v*) digested or undigested colostrum in a fresh medium or left untreated (fresh medium only) for one hour. Then 10 µL of lipopolysaccharides (LPS) from *Escherichia coli* O111:B4 (5 µg mL^−1^; Sigma Aldrich, St. Louis, MO, USA) or recombinant human tumor necrosis factor (TNF) (100 ng mL^−1^; Peprotech, Cranbury, NJ, USA) was added to the culture for overnight stimulation. The cells that remained untreated were set as controls.

### 2.4. Measurements of IL-8 and Cytotoxicity

Following overnight stimulation, the supernatant from each well was transferred to microcentrifuge tubes and span at 1000× *g* for 10 min. Interleukin-8 (IL-8; marker of inflammation) was measured using Enzyme Linked Immunosorbent Assay (ELISA; Human IL-8/CXCL8 DuoSet; R&D Systems, Minneapolis, MN, USA) within the detection range of 31.2 to 2000 pg mL^−1^. The absorbance of each well at 450 nm was measured by a microplate reader (SpectraMax i3x, Molecular Devices, San Jose, CA, USA). Cytotoxicity was examined by measuring the release of lactate dehydrogenase (LDH) from the cell supernatants using the CyQUANT^TM^ LDH Cytotoxicity Assay kit (Invitrogen, Waltham, MA, USA). The absorbance of each well at 490 nm was measured. Percent cytotoxicity was calculated as: (Treatment LDH activity − Control LDH activity)/(Maximum LDH activity − Control LDH activity) × 100.

### 2.5. Statistical Analysis

All analyses were performed with GraphPad Software Prism 9.3.1 (GraphPad Software, Inc., San Diego, CA, USA). Results were shown as mean ± standard error (SEM). Data were analyzed through one-way analysis of variance (ANOVA) followed by Tukey’s multiple comparisons. *p*-value of <0.05 was set as the significant cutoff.

## 3. Results

### 3.1. LPS and TNF Stimulation Induced IL-8 Production

**Figure 1:**
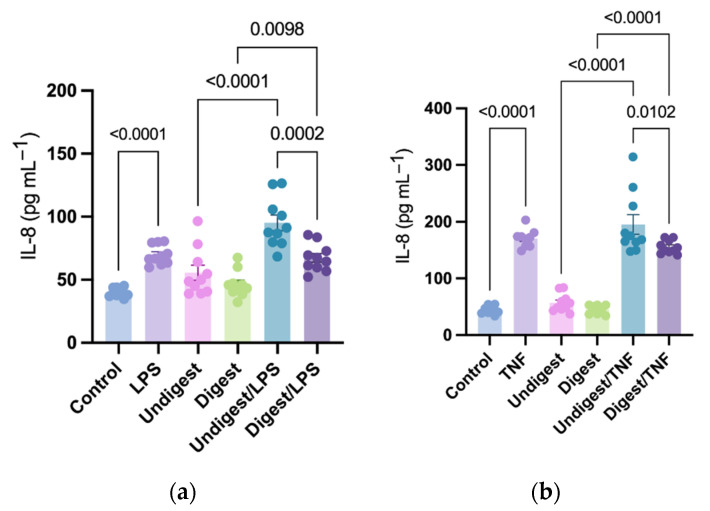
Production of interleukin-8 in human intestinal epithelial (Caco2BBe) cells in response to lipopolysaccharides (**a**) and tumor necrosis factor (**b**) stimulation after pretreatment with digested and undigested colostrum. Data represented are mean ± standard error (SEM) from nine to ten human colostrum samples (outliers removed). Data were analyzed through one-way analysis of variance (ANOVA) followed by Tukey’s multiple comparisons. IL-8 = interleukin-8; LPS = lipopolysaccharides; TNF = tumor necrosis factor.

LPS and TNF significantly induced IL-8 production compared with control (69.82 ± 2.44 vs. 39.99 ± 1.14 pg mL^−1^; *p* < 0.0001 and 170.7 ± 5.29 vs. 44.05 ± 2.11 pg mL^−1^; *p* < 0.0001, respectively; Figure 1a,b). The amount of TNF-induced IL-8 was significantly higher than that induced by LPS.

### 3.2. Digested Colostrum Reduced IL-8 Production in Caco2BBe Cells under Both LPS and TNF Stimulation

Without stimulation, pretreatment with digested colostrum showed no difference compared with the undigested groups (*p* > 0.05 in all comparisons, Figure 1a,b). Cells pretreated with both digested and undigested colostrum produced a higher amount of IL-8 under LPS stimulation compared with their unstimulated counterparts (67.41 ± 3.45 vs. 46.51 ± 3.25 pg mL^−1^; *p* = 0.0098 and 95.18 ± 6.18 vs. 55.61 ± 5.99 pg mL^−1^; *p* < 0.0001, respectively; Figure 1a). Similarly, under TNF stimulation, cells pretreated with digested and undigested colostrum produced a higher level of IL-8 compared with their unstimulated counterparts (*p* < 0.0001 in all comparisons, Figure 1b). Compared with undigested groups, the digested colostrum significantly reduced IL-8 production under both LPS stimulation (95.18 ± 6.18 vs. 67.41 ± 3.45 pg mL^−1^; *p* = 0.0002; Figure 1a) and TNF stimulation (195.30 ± 17.36 vs. 155.50 ± 3.64 pg mL^−1^; *p* = 0.0102; Figure 1b).

### 3.3. The Inhibition Effects on IL-8 Production Varies among Individual Human Colostrum

**Figure 2:**
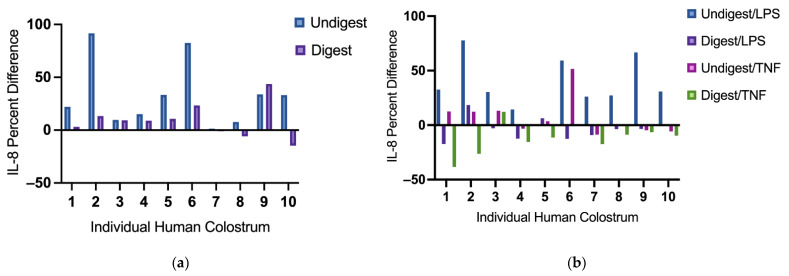
Interleukin-8 difference in individual digested and undigested human colostrum in response to no stimulus (**a**) and stimuli (lipopolysaccharides or tumor necrosis factor) (**b**). Percent difference was calculated to identify the change of IL-8 production in Caco2BBe cells pretreated with digested or undigested colostrum against their unpretreated counterparts. It was calculated as: (Pretreated IL-8 production−Unpretreated IL-8 production)/Unpretreated IL-8 production × 100. From left to right: human colostrum sample 1–10. IL-8 = interleukin-8; LPS = lipopolysaccharides; TNF = tumor necrosis factor.

Without stimulation, most digested colostrum samples reduced IL-8 production compared with their undigested counterparts (Figure 2a). Under LPS stimulation, IL-8 production was suppressed following pretreatment with nearly all digested colostrum samples (except sample 2 and 5), but not with undigested colostrum samples (Figure 2b). Under TNF stimulation, variations were also observed among these samples; all but one digested colostrum sample (sample 3) reduced IL-8 production. Undigested colostrum sample 4, 7, 9, 10 inhibited IL-8 production (Figure 2b). Among samples that suppressed IL-8 production, the extent of suppression differed (Figure 2a,b).

### 3.4. Digested and Undigested Colostrum Did Not Affect Cytotoxicity in Caco2BBe Cells Overall

**Figure 3:**
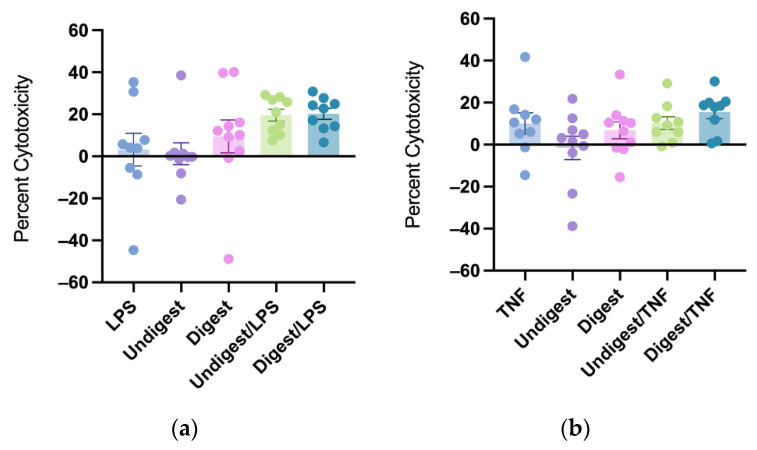
Percent cytotoxicity in response to lipopolysaccharides (**a**) and tumor necrosis factor (**b**) after pretreatment with digested and undigested colostrum. Percent cytotoxicity was examined by the release of lactate dehydrogenase (LDH) calculated as: (Treatment LDH activity − Control LDH activity)/(Maximum LDH activity − Control LDH activity) × 100. Data represented are mean ± standard error (SEM) from nine to ten colostrum samples (outliers removed). Data were analyzed through one-way analysis of variance (ANOVA) followed by Tukey’s multiple comparisons. LPS = lipopolysaccharides; TNF = tumor necrosis factor.

Neither digested nor undigested colostrum significantly affected cytotoxicity under both LPS and TNF stimulation. There was no statistical difference between digested and undigested groups on cytotoxicity levels (Figure 3a,b).

### 3.5. Individual Colostrum Affected Cytotoxicity Differently

**Figure 4:**
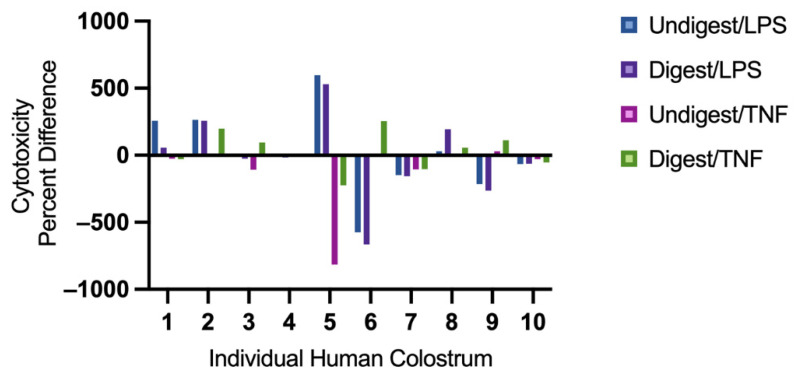
Cytotoxicity percent difference in individual digested and undigested human colostrum in response to lipopolysaccharides or tumor necrosis factor. Cytotoxicity percent difference was calculated to identify change of percent cytotoxicity in Caco2BBe cells following treatment of digested or undigested colostrum samples against their unpretreated controls. It was calculated as: (Pretreated percent cytotoxicity − Unpretreated percent cytotoxicity)/Unpretreated percent cytotoxicity × 100. From left to right: human colostrum sample 1–10. LPS = lipopolysaccharides; TNF = tumor necrosis factor.

As shown in Figure 4, the extent of cytotoxicity differed among colostrum samples. Colostrum 2 and 8 induced cytotoxicity in all conditions; colostrum 3, 4, 7 and 10 reduced cytotoxicity in all conditions (except for digested colostrum 3 under TNF stimulation); colostrum 6 and 9 reduced cytotoxicity under LPS stimulation, but increased cytotoxicity under TNF stimulation; colostrum 1 reduced cytotoxicity under TNF stimulation, but increased cytotoxicity under LPS stimulation.

## 4. Discussion

In this study, we compared the protective effects of digested colostrum versus undigested colostrum against LPS- and TNF-stimulations. We found that pretreatment with digested colostrum significantly reduced both LPS and TNF-induced inflammation in human intestinal epithelial cells, while not affecting cytotoxicity. We also observed widely variable differences in anti-inflammatory effects among individual colostrum samples.

In this work, we optimized the dose of LPS in Caco2BBe cells that would induce a robust response to allow adequate margin for IL-8 reduction (data not shown). In addition to the previous finding that digested colostrum suppressed TNF-induced IL-8 production [12], we found that it also suppressed IL-8 production after exposure to LPS. Its significant anti-inflammatory effect suggests that the digestion process may have further released additional bioactive components to exert anti-inflammatory effects on induced cells compared with undigested colostrum. One major class of products released from digestion are peptides with partially known bioactivities [14,15,16,17,18] (e.g., antimicrobial, anti-inflammatory, angiotensin-converting-enzyme (ACE)-inhibitory), whereas when encrypted as part of the intact milk proteins, such as the form found in the undigested milk, are inactive [7,19]. This might explain why digested colostrum provided more protection in this study. Our results suggest that potential bioactive peptides from the digestion can protect the intestinal epithelial cells by reducing LPS-induced inflammation. As a cell component of gram-negative bacteria, LPS can interact with the toll-like receptor 4 (TLR-4) from intestinal enterocytes, trigger cascades of signaling and inducing a severe proinflammatory response, especially in premature infants with underdeveloped intestines which can develop NEC [20]. Our results add insight into the protection of human milk against NEC from the perspective of the digestion-released bioactive peptides, as they are presumed to be functional during their passage through the infant GI tract [21], even at the ileocecal region where NEC occurs. Although it remains unclear how human milk and colostrum-derived peptides affect the infant intestinal mucosa during their release along the GI tract, new work is underway to further explore this area of research [22].

While not as robustly nor as consistently as digested colostrum, some undigested samples did decrease IL-8 concentrations. This suggests that endogenous bioactive components may inhibit inflammation [23,24,25,26,27]. This corresponds to the fact that human colostrum originally contains a number of bioactive factors in higher amounts that can modulate the inflammatory response, such as anti-inflammatory cytokines (e.g., interleukin-10 (IL-10)) [26], bioactive proteins (e.g., lactoferrin) [24], human milk oligosaccharides (HMOs) [25], immunoglobulins (e.g., secretory immunoglobulin-A (sIgA)) [23], as well as bioactive peptides released from mammary gland digestion [27]. While most will end up being degraded, some human milk factors remain intact during passage through the infant gut due to their resistance toward digestion, the existence of anti-proteolytic enzymes in colostrum, as well as the less developed digestion system in preterm infants [28,29,30]. Nonetheless, we showed that digested colostrum suppressed IL-8 production significantly more than undigested samples warranting future investigations to identify the differences in bioactive constituents between the two.

Consistent with the previous work, we observed a wide variation on the extent to which these colostrum samples exerted anti-inflammatory effects under all conditions. This is expected as biologic differences (both nutritional [31,32] and immunological components [33,34]) in human milk from different mothers have been widely reported. Yet, how individual human milk variances affect the intestinal health of infants remains unknown. However, as reported in NEC occurrence, some exclusively human milk fed infants can still develop NEC [35]. This corroborates with our hypothesis that individual human milk that harbors certain profile of bioactive components may possess higher potency to lower the risk of intestinal inflammation and development of NEC while some may not. 

To our knowledge, how human milk affects the cytotoxicity in human intestinal epithelial cells is not fully described. Scarce evidence showed that storage conditions of human milk at a donor, milk bank or research lab can cause initial or additional cytotoxicity. The activity of lipase in milk which produces free fatty acids can also lead to increased toxicity to epithelial cells [36]. In this study, the pooled data on digested and undigested colostrum did not show a significant inducing or suppressing effect on cytotoxicity under LPS and TNF stimulation. However, it is important to note that we optimized the dose of LPS and TNF and stimulation time to best identify IL-8 production. Since cytotoxicity was measured downstream from the LPS/TNF stimulation, it might not be optimal for detecting LDH cytotoxicity in this study. However, while not statistically significant, the individual colostrum samples exhibited large variations on cytotoxicity. This can still be attributed to the biological difference among these individual colostrum samples. Future study is necessary to further understand the impact of human milk components on cytotoxicity. 

To our knowledge, this is the first study to compare the anti-inflammatory effects of digested and undigested human colostrum from different mothers on induced human intestinal epithelial cells. The digested colostrum shows its protection against inflammation, which brings digestion-released bioactive components into focus. To further identify the effects of these bioactive compounds, a more robust cellular model is needed to overcome the limitation of using one single intestinal cell type in this work. Future study will incorporate the enteroids model (a 3D cell culture that recapitulates the complexity of in vivo intestinal epithelium and consists of all differentiated intestinal epithelial cell types) [37]. Additionally, we will identify the anti-inflammatory fractions from individual colostrum samples (such as specific bioactive peptides) and further characterize their functions and underlying protecting mechanisms to develop a better understanding on infant gastrointestinal health.

## 5. Conclusions

In this study, we identified the effect of digested colostrum versus undigested colostrum on inflammation and cytotoxicity in human intestinal epithelial cells under LPS and TNF stimulation. Our primary finding is that in vitro-digested human colostrum suppressed an inflammatory response more than undigested human colostrum. Our results add insights into the bioactive potential of human milk following digestion on infant gut health. Despite our findings, there are limitations in this study. Future work will overcome these limitations by increasing the human milk sample size, incorporating advanced cell culture models that recapitulate the gut environment, and adding measurements on a variety of proinflammatory markers.

## Data Availability

Not applicable.
